# Genetic diversity of native and cultivated Ugandan Robusta coffee (*Coffea canephora* Pierre ex A. Froehner): Climate influences, breeding potential and diversity conservation

**DOI:** 10.1371/journal.pone.0245965

**Published:** 2021-02-08

**Authors:** Catherine Kiwuka, Eva Goudsmit, Rémi Tournebize, Sinara Oliveira de Aquino, Jacob C. Douma, Laurence Bellanger, Dominique Crouzillat, Piet Stoffelen, Ucu Sumirat, Hyacinthe Legnaté, Pierre Marraccini, Alexandre de Kochko, Alan Carvalho Andrade, John Wasswa Mulumba, Pascal Musoli, Niels P. R. Anten, Valérie Poncet

**Affiliations:** 1 Department of Plant Sciences, Centre for Crop Systems Analysis, Wageningen University & Research (WUR), Wageningen, The Netherlands; 2 National Agricultural Research Organization (NARO), Entebbe, Uganda; 3 IRD—UMR DIADE (Univ. Montpellier, CIRAD, IRD), Montpellier, France; 4 EMBRAPA Coffee-INOVACAFE, Lavras, Brazil; 5 Nestlé Research, Tours, France; 6 Meise Botanic Garden, Meise, Belgium; 7 ICCRI, Jember, Indonesia; 8 CNRA, Divo, Côte d’Ivoire; 9 Institute of Agricultural Genetics (AGI)—LMI RICE2, CIRAD—UMR IPME (Univ. Montpellier, CIRAD, IRD), Hanoi, Vietnam; Chinese Academy of Sciences, CHINA

## Abstract

Wild genetic resources and their ability to adapt to environmental change are critically important in light of the projected climate change, while constituting the foundation of agricultural sustainability. To address the expected negative effects of climate change on Robusta coffee trees (*Coffea canephora*), collecting missions were conducted to explore its current native distribution in Uganda over a broad climatic range. Wild material from seven forests could thus be collected. We used 19 microsatellite (SSR) markers to assess genetic diversity and structure of this material as well as material from two *ex-situ* collections and a feral population. The Ugandan *C*. *canephora* diversity was then positioned relative to the species’ global diversity structure. Twenty-two climatic variables were used to explore variations in climatic zones across the sampled forests. Overall, Uganda’s native *C*. *canephora* diversity differs from other known genetic groups of this species. In northwestern (NW) Uganda, four distinct genetic clusters were distinguished being from Zoka, Budongo, Itwara and Kibale forests A large southern-central (SC) cluster included Malabigambo, Mabira, and Kalangala forest accessions, as well as feral and cultivated accessions, suggesting similarity in genetic origin and strong gene flow between wild and cultivated compartments. We also confirmed the introduction of Congolese varieties into the SC region where most Robusta coffee production takes place. Identified populations occurred in divergent environmental conditions and 12 environmental variables significantly explained 16.3% of the total allelic variation across populations. The substantial genetic variation within and between Ugandan populations with different climatic envelopes might contain adaptive diversity to cope with climate change. The accessions that we collected have substantially enriched the diversity hosted in the Ugandan collections and thus contribute to *ex situ* conservation of this vital genetic resource. However, there is an urgent need to develop strategies to enhance complementary *in-situ* conservation of *Coffea canephora* in native forests in northwestern Uganda.

## Introduction

Coffee is a major global commodity and the total value of its industry was estimated to surpass US$200 billion in 2017 [1]. The coffee industry is mainly (99%) underpinned by two *Coffea* species, i.e. *Coffea arabica* and *C*. *canephora* [2]. Uganda accounts for 7% of global *C*. *canephora* exports and the whole coffee sector provides a livelihood for about 8 million people [3]. The sustainability of Ugandan *C*. *canephora* production is thus of major national and global importance, particularly for smallholder farmers [4]. Unfortunately, the sustainability of the global coffee industry is threatened by the adverse effects of climate change, especially drought, rising temperatures, pest and disease pressure [2,5,6]. A global temperature increase of 2.1° C has been predicted by 2050 [7], while rainfall is expected to become more erratic, with more frequent and severe drought periods, which will render conditions in some coffee growing areas less suitable, seriously affecting coffee production [8–11].

The ability of crops to adapt to environmental challenges, such as the effects of climate change, depends on the extent of genetic variation within the crop. The resilience potential of all crop production systems is anchored by the intraspecific trait diversity that has evolved in the species’ natural habitat [12]. There has been a steady increase in the use of crop wild relatives (CWR) to improve the adaptive potential and resistance of crops to pests and diseases, as noted in wheat and tomato whose improved cultivars include genes from their wild relatives [13]. As the effects of climate change set in, the use of congeneric and conspecific wild coffee variants is becoming of primary importance to confer tolerance and resilience to *C*. *arabica* and *C*. *canephora* [14,15]. The genetic diversity that exists within natural habitats needs to be explored to promote the use of wild variants for coffee improvement.

*Coffea canephora* is a diploid (2n = 2x = 22), self-incompatible [16] insect- and wind pollinated [17] species. Wild coffee seed dispersal is facilitated by frugivorous animals, in Uganda primarily by monkeys and bats [18]. *C*. *canephora* natural distribution range in Africa stretches from Guinea in the west to Uganda in the east and Angola in the south [19]. Among *Coffea* species, *C*. *canephora* has the widest distribution and climatic range *e*.*g* rainfall levels differ by 3-fold across the species’ distribution range. Development of molecular markers such as SSR (single sequence repeat), RFLP (restriction fragment length polymorphism) and RAPD (random amplification of polymorphic DNA) markers, or more recently SNPs, derived from DNA sequencing has enabled researchers to further understand the phylogenetic relationships between different *Coffea* species [20,21], but also the relationships within wild *C*. *canephora* populations [22,23]. Genetic characterization of *C*. *canephora* diversity has been greatly facilitated by the availability of a large repository of simple sequence repeat (SSR) based microsatellite markers, which provides efficiency and high-resolution in genetic analyses [24–28]. Globally, the genetic diversity of *C*. *canephora* is linked to the geographical location [22,23,29,30]. Isoenzyme analyses initially highlighted that *C*. *canephora* diversity was pooled within two different genetic groups, namely: (i) a Guinean group including wild populations from Côte d’Ivoire, and (ii) a Congolese group consisting of accessions from the Central African Republic and Cameroon [30]. Using RFLP and SSR markers, Gomez et al. [22] pooled *C*. *canephora* genetic diversity into five genetic groups (A, B, C, D and E). Geographically, genetic group A comprised wild populations from Congo and Cameroon, group B from eastern-central Africa, group C from western-central Africa, Cameroon and northeastern Congo, group E from Congo and southern Cameroon, while group D consisted of wild populations from Côte d’Ivoire and Guinea. Musoli et al. [31] further determined that some Ugandan wild populations clustered into another distinct group (group O). Finally, Merot-L’Anthoene et al. [23], using a genome-wide Coffee 8.5K SNP array, described *C*. *canephora* genetic diversity with eight distinct genetic groups, including the Ugandan one (group O), thus identifying two new genetic groups, i.e. R (comprising samples from southern Democratic Republic of the Congo) and G (comprising samples from Angola), whereas the differentiation between groups E and R was weaker (S1 Fig).

Uganda lies within a dry geographical area of the *C*. *canephora* distribution range and the wild populations occur in five climatic zones with different rainfall levels, as described by [32]. Wild Ugandan *C*. *canephora* populations therefore likely differentially evolved in respect to the environmental gradient. This suggests that there is substantial genetic variation within and across Ugandan populations that could be explored for its functional importance relative to climate change and other production challenges. Efforts to unravel Ugandan *C*. *canephora* genetic diversity have been partially accomplished by [31], who reported that wild and cultivated individuals were clearly delineated and that genetic diversity (allelic richness and heterozygosity) was higher in cultivated than in wild compartments. However, these authors only studied two wild populations (from Itwara and Kibale forests) and thus did not include the whole range of wild Ugandan populations and did not link the genetic variation to the environmental gradient. In the present study, we collected samples representing most of the present day native distribution of *C*. *canephora* in Uganda across a broad climatic range and we also included material from *ex-situ* collections that underpin the on-going Ugandan coffee breeding program. We aimed to decipher the genetic diversity, population structure of Ugandan *C*. *canephora*, while characterizing the environmental envelopes that delineate the population distributions across the whole geographical range. We more specifically aimed to: (i) determine the level of genetic diversity, population structure and genetic relationships between wild, feral (formerly cultivated material returned to wild) and cultivated *C*. *canephora* genotypes, (ii) position Ugandan *C*. *canephora* diversity relative to the species’ global diversity structure, and (iii) identify the relationship between the genetic structure of Ugandan *C*. *canephora* wild populations with their climatic profiles.

## Materials and methods

### Study area and field sampling

**Fig 1:**
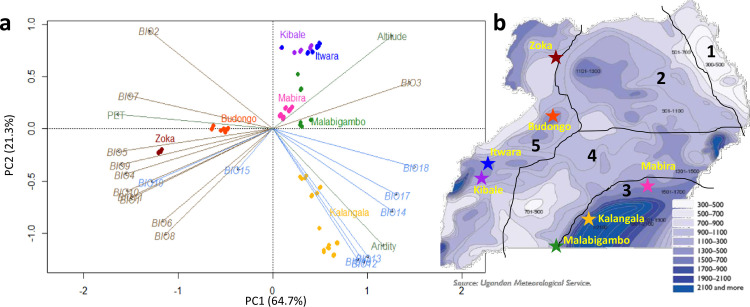
Environmental context of the sampled populations. a- Principal component analysis (PCA) of 22 environmental variables at Ugandan *C*. *canephora* native sites. For the BIOx (see S3 Table), temperature-related variables are shown in grey-brown; precipitation-related in blue. Elevation, aridity index (AI) and potential evapotranspiration (PET) are shown in green. The first two axes, *i*.*e*. PC1 and PC2, account for 64.7% and 21.3% of the total variation, respectively. b- The seven natural forests are reported on the Ugandan map of average annual rainfall from the Ugandan Meteorological Service (NEMA, 2009) [34]. The five climatic zones based on precipitation [Matete et al. 2010] [33] are delimitated in grey. They include: 1. Karamoja, 2. Acholi-Kyoga, 3. Lake. Victoria, 4. Ankole-Southern Uganda and 5. Western Uganda areas. Climatic zones:3, 4 and 5 are important with regard to the presence of wild and cultivated of *C*. *canephora* populations. *National Environment Management Authority (NEMA)*. *2009*. *Uganda*: *Atlas of our changing environment*. *NEMA*, *Kampala*, *Uganda*.

Based on annual precipitation records, Uganda was broadly categorized in five distinct climatic zones [32–34], namely: (i) Karamoja (500–750 mm, annual rainfall range), (ii) Acholi (750–1,000 mm), Lake Victoria (1,000–1,500 mm), southern Ankole (1,500–2,000 mm) and western Uganda (> 2,500 mm). Three of the five broad climatic zones: Lake Victoria, southern Ankole and western Uganda are important with regard to the presence of wild and cultivated of *C*. *canephora* populations. The hierarchical sampling strategy described by [31] was applied to collect samples representing the *C*. *canephora* distribution in these three distinct climatic zones. Wild samples were collected in 2015 and geo-referenced from seven natural forests: Zoka, Budongo, Itwara, Kibale, Mabira, Malabigambo and Lutoboka central forest reserve on Kalangala main Island (Fig 1B). These forests are protected and permission to access and collect study samples was obtained from the National Forestry Authority (NFA; NFA/N/9.2/14) and the Uganda Wildlife Authority (UWA; EDO/35/01). In each targeted forest, samples were collected from five sub-sites separated by distances of at least 5 km. At each sub-site, a minimum of five healthy trees was selected for collection of leaves for DNA extraction. Of these same trees, cuttings were made for *ex-situ* conservation at the National Coffee Resources Institute (NaCORI) in Kituza and the Plant Genetic Resources Centre (PGRC) in Entebbe. In addition, individuals described as feral (formerly cultivated, abandoned for over 50 years and returned to wild) were collected from Kalangala main Island, Bunyama and Bugala islands of Kalangala district in Lake Victoria. The feral samples were collected from six different sub-sites separated by 10 km and located at least 0.5–2 km from the edges of the Islands. The Kalangala study site thus included both wild and feral populations. The cultivated set of individuals was represented by a total of 52 samples collected from the two germplasm field collections of the Ugandan National Agricultural Research Organization (NARO): 32 samples from the National Coffee Research Institute Kituza and 20 samples from the National Agricultural Research Laboratories at Kawanda. The cultivated genotypes were selected on the basis of their historical and passport data with the aim of representing the total range of traditional and commercially cultivated *C*. *canephora* diversity, including the two predominant forms found in Uganda: Erecta, or upright forms, and Nganda, or spreading forms [35] and the six elite clones, namely: KW13, KW14, KW15, KW16, KW18 and KW19 (details can be found in S1 Table). The number of accessions per collection site (269 individuals in total) is presented in Table 1. We assessed the genetic position of these Ugandan *C*. *canephora* accessions within the overall species diversity by comparing them with a representative set of wild accessions from the *C*. *canephora* diversity groups, as previously defined with SSR markers or the SNP array [22,23] (S2 Table).

**Table 1:** Ugandan *C*. *canephora* sample origin. Mean and standard deviation of selected environmental variables across locations. All variables were significantly different across locations based on a one-way ANOVA (p-value < 2.2e-16). Pairwise *t*-test results between locations are also presented.

	Sample number			Location			Annual mean temperature (°C)	Annual precipitation (mm)	Potential of evapotranspiration	Aridity Index
Location	Wild	Feral	Cultivated	Latitude	Longitude	Elevation (m.a.s.l.)	BIO1	BIO12	PET	AI
**Zoka**	46			3.127 ± 0.004	31.655 ± 0.003	929 ± 12.9 ^a^	24.43 ± 0.5 ^a^	1266.23 ± 2.4^e^	1868.52 ± 0.7 ^a^	0.68 ± 0.00 ^a^
**Budongo**	54			1.765 ± 0.105	31.544 ± 0.121	1057 ± 35.1 ^b^	23.13 ± 2.4 ^b^	1317.60 ± 40.0^d^	1740.74 ± 13.7 ^b^	0.76 ± 0.03 ^b^
**Itwara**	23			0.786 ± 0.082	30.437 ± 0.014	1491 ± 47.0 ^c^	19.76 ± 3.0 ^c^	1421.83 ± 55.0^b^	1603.87 ± 13.0 ^c^	0.89 ± 0.04 ^c^
**Kibale**	19			0.498 ± 0.060	30.460 ± 0.025	1367 ± 47.8 ^d^	20.43 ± 3.8 ^d^	1267.00 ± 63.3^e^	1636.50 ± 17.6 ^d^	0.77 ± 0.05 ^b^
**Malabigambo**	16			-0.892 ± 0.019	31.715 ± 0.056	1149 ± 8.8 ^e^	20.94 ± 1.6 ^e^	1414.31 ± 80.5^b^	1604.44 ± 6.3 ^c^	0.88 ± 0.05 ^c^
**Mabira**	23			0.448 ± 0.021	33.013 ± 0.063	1195 ± 32.0 ^f^	21.64 ± 1.8 ^f^	1356.20 ± 20.8^c^	1651.84 ± 11.8 ^e^	0.82 ± 0.02 ^d^
**Kalangala**	10	26		-0.307 ± 0.041	32.223 ± 0.075	1189 ± 34.1 ^f^	21.41 ± 1.7 ^g^	1941.70 ± 170.9^a^	1559.78 ± 11.4 ^f^	1.25 ± 0.12 ^e^
**Kituza collection**			32							
**Kawanda collection**			20							
**Total**	**191**	**26**	**52**							

BIO1, annual mean temperature (°C); BIO12, Annual precipitation (mm); PET, Potential of evapotranspiration; Elevation (m.a.s.l., meter above sea level); AI, Aridity Index (see S3 Table). a-f per column, a different letter means that the results are significantly different according to the pairwise t-test with a 5% level of significance.

### DNA isolation and genotyping assay

Silica-dried leaves from the 269 study samples were ground in liquid nitrogen with a mortar and pestle. DNA was extracted using the DNeasy® Plant Maxi kit (QIAGEN), with few adjustments to tailor it for dry leaf material. Extractions were performed by adding double amounts of both AP1 and P3 buffers. Nuclear genetic variation was assessed at 19 SSR loci (S3 Table) using markers developed at R&D Nestlé Tours and selected based on their capacity to discriminate *C*. *canephora* genotypes. The multiplex PCRs were carried out using the QIAGEN Type-it® Microsatellite PCR kit (Ref 206246), in a 25-μL reaction mixture containing the “Master Mix”, 50 ng template DNA, 0.2 μM of each reverse and fluorescence-labeled primers. The PCR amplification was performed using the following cycling conditions: initial denaturation at 95°C for 5 min, followed by 28 cycles of denaturation at 95°C for 30 s, annealing at 60°C for 1.5 mim, and extension at 72°C for 30 s, followed by a final extension at 60°C for 30 min. Subsequently, ABI 3500 XL sequencer (Life Technologies, Foster City, CA, USA) was used to analyze the PCR products, and their sizes (bp) were determined using GeneMapper® version 6 (Applied Biosystems, USA). The PCR amplification conditions and all information on the markers are also given in MoccaDB, an integrative database for functional, comparative and diversity studies on the Rubiaceae family (http://moccadb.ird.fr/ [23,36]) and in the coffee genome hub (http://coffee-genome.org/ [37]).

### Data analysis

#### Genetic analysis

Within-population indices of genetic diversity such as the number of alleles (Na), number of effective alleles (Ne), number of private alleles (P, number of alleles unique to a single population in the data set), observed and expected heterozygosity (H_*o*_ and H_*e*_) and inbreeding coefficient (fixation index, F) were estimated for each sampled locality using GenAlEx software version 6.5 [38]. while allelic richness (Rs) was estimated using FSTAT version 2.9.4 [39]. GenAlEx software was also used to compare the populations by calculating the genetic differentiation among populations (F_*st*_, [40]).

The genetic structure of the diversity was analyzed using the Bayesian clustering method of the STRUCTURE version 2.3.4 software package. This model-based clustering method uses a Bayesian approach to detect underlying genetic (sub)-populations within a group of individuals that are genotyped with multiple markers [41]. Furthermore, for each genotype, the program highlights the proportion of the genome originating from the inferred populations [41]. The most likely number of genetic clusters was estimated for the wild individuals without feral/cultivated samples based on the methods described in [42]. The default settings of the analysis were as follows: “admixture model” and “allele frequencies correlated”. Each run was performed during 100,000 Markov Chain Monte Carlo iterations with a burn in of 100,000 iterations.

To investigate the genetic relationships between accessions, either at the species level (including representative set of groups defined by [21] (S2 Table, S1 Fig) or within the Ugandan sample set (wild samples with or without feral/cultivated individuals), genetic dissimilarity matrices were computed in DARwin software version 6 [43]. The dissimilarity between samples was calculated by using simple matching based on the Sokal and Michener index [44]. The dissimilarity formula is:

$d_{ij}=1-\frac{1}{L}\sum_{I=1}^{L}\frac{m_{l}}{\pi }$, where *dij* is the genetic dissimilarity between units *i* and *j*, *L* is the number of loci and *ml* is the number of matching alleles for locus *l*. π represent the ploidy of the organisms.

Dissimilarities were used for the construction of unweighted neighbour-joining trees in DARwin software version 6 [43].

#### Climate characterization of Uganda’s C. canephora native sites

Twenty-two environmental variables were retrieved for each georeferenced wild sample and used as predictors to characterize the climatic characteristics of each study site. Nineteen out of the 22 environmental variables were taken from a global climate database (WorldClim, see below), and the three others were: elevation, aridity index (AI) and potential evapotranspiration (PET: a measure of the ability of the atmosphere to remove water through evapotranspiration) (S4 Table). These variables were selected as they encompass primary climate factors and variation, so they have an important impact on the ecophysiology of the species [45]. The 19 bioclimatic variables averaged for the years 1950–2000 were downloaded from WorldClim (www.worldclim.org) at 30 arc-second resolution [46]. Elevation data was collected in the field using a Garmin eTrex 10 GPS device. Aridity index (AI: which quantifies the availability of precipitation over atmospheric water demand), and PET data were sourced from the Global-Aridity database [47]. AI values were estimated as a ratio of mean annual precipitation to the mean annual potential evapotranspiration [47]. PET values were calculated using the mean monthly temperature, mean monthly temperature range and mean monthly extraterrestrial radiation [48]. The formula for AI and PET are:

$$AI=\frac{MAP}{MAE}$$

where *AI* = aridity index, *MAP* = mean annual precipitation, *MAE* = mean annual potential evapotranspiration. and

$$PET=0.0023*RA*(T_{mean}+17.8)*{TD}^{0.5}(mm/d)$$

where: *PET* = potential evapotranspiration, *RA* = mean monthly extraterrestrial radiation, *T*_*mean*_ = mean monthly temperature, *TD* = mean monthly temperature range.

Since the locations had unequal sample sizes, we performed Welch’s one-way tests to assess whether there were significant differences in the means of the selected environmental variables across locations. We subsequently performed pairwise *t*-tests with no assumption of equal variances to determine if the mean differences between specific pairs of locations were statistically significant. Moreover, Pearson correlation coefficients were calculated to test the relationship between all of the studied environmental variables. All tests were deemed significant at *p* ≤ 0.05 and the analysis was performed with R software version 3.5.0 [49].

#### Analysis of relationships between the climatic profiles and the genetic diversity

A principal component analysis (PCA) was performed on the 22 above-mentioned environmental variables to describe the variation in environmental conditions across sites. Furthermore, a redundancy analysis (RDA) was conducted to investigate the amount of genetic variation that could be explained by the environmental conditions at the study sites [50]. Then forward and backward selection was carried out and only significant environmental variables were kept. PCA and RDA analyses were performed in the “vegan” library in R software version 3.5.0 [50]. To test whether there was isolation by distance (IBD) between each native individual, a Mantel test with 999 permutations was performed to assess the correlation (linear) between the genetic distance (calculated as described by [51]) and the geographical distance (from decimal degrees) implemented in GenAlex version 6.5 [38] and the correlation was reported as significant if *p* ≤ 0.01.

## Results

### Genetic diversity and structure of Ugandan wild C. canephora populations

The genetic diversity in Ugandan *C*. *canephora* native populations was examined at the 19 SSR loci and in the 191 *C*. *canephora* accessions collected from wild populations (Table 2). An average of 4.7 alleles per locus (Na) were detected over the native populations. Populations from Itwara and Kibale had the lowest allelic richness (Rs of 2.88 and 2.96, respectively) and heterozygosity (H_*e*_ of 0.45 and 0.41, respectively). In contrast, the population from Mabira had the highest allelic richness (Rs = 4.75) and heterozygosity (H_*e*_ = 0.62) among the wild populations. Notably, populations from Zoka and Budongo, with a relatively high allelic richness (Rs = 4.43 and 3.82, respectively) and expected heterozygosity (H_*e*_ = 0.58 for both), appeared to be relatively unique among the study populations. They had the highest average number of private alleles across all loci (0.6 and 0.3, respectively) compared to Itwara, Kibale or Malabigambo with all less than 0.1.

**Table 2:** Allelic pattern across populations. Values are provided for each native forest, the feral population and the two collections: Number of alleles, number of effective alleles, number of private alleles, allelic richness, observed and expected heterozygosities, and fixation index. Since Mabira, Malabigambo and Kalangala belong to the same south-central genetic group [SC], data are also provided for this group.

Forests	Status	N	Na	Ne	Pv	Rs	H_*o*_	H_*e*_	F
**Zoka**	Wild	46	5.7	3.3	0.6	4.43	0.51	0.58	0.1
**Budongo**	Wild	54	5.1	3	0.3	3.82	0.52	0.57	0.07
**Itwara**	Wild	23	3.1	2.2	0.0	2.88	0.41	0.45	0.11
**Kibale**	Wild	19	3.3	2.1	0.1	2.96	0.33	0.41	0.2
**[SC] Mabira**	Wild	23	5.9	3.3	0.1	4.75	0.57	0.62	0.08
**[SC] Malabigambo**	Wild	16	4.8	2.9	0.1	4.29	0.56	0.55	-0.01
**[SC] Kalangala**	Wild	10	4.7	2.9	0.0	4.68	0.64	0.58	-0.1
	**SC group**	49	6.5	3.3	0.2		0.58	0.62	0.06
	**Mean over wild pop.**		**4.7**	**2.8**	**0.2**	**3.97**	**0.51**	**0.54**	**0.06**
**Kalangala**	Feral	26	5.6	3.3	0.1	4.48	0.62	0.61	0.0
**Kituza**	Collection	32	6.1	3.5	0.2	4.70	0.6	0.63	0.04
**Kawanda**	Collection	20	5.5	3.6	0.0	4.78	0.63	0.63	-0.02

N = sample size, Na = average No. of different alleles, Ne = No. of effective alleles = 1/(Sum pi^2); Pv = average No. of private alleles across all loci, Rs = allelic richness based on min. sample size of 10 diploid individuals, H_*o*_ = expected heterozygosity, H_*e*_ = expected heterozygosity = 1—Sum pi^2, F = fixation index.

**Fig 2:**
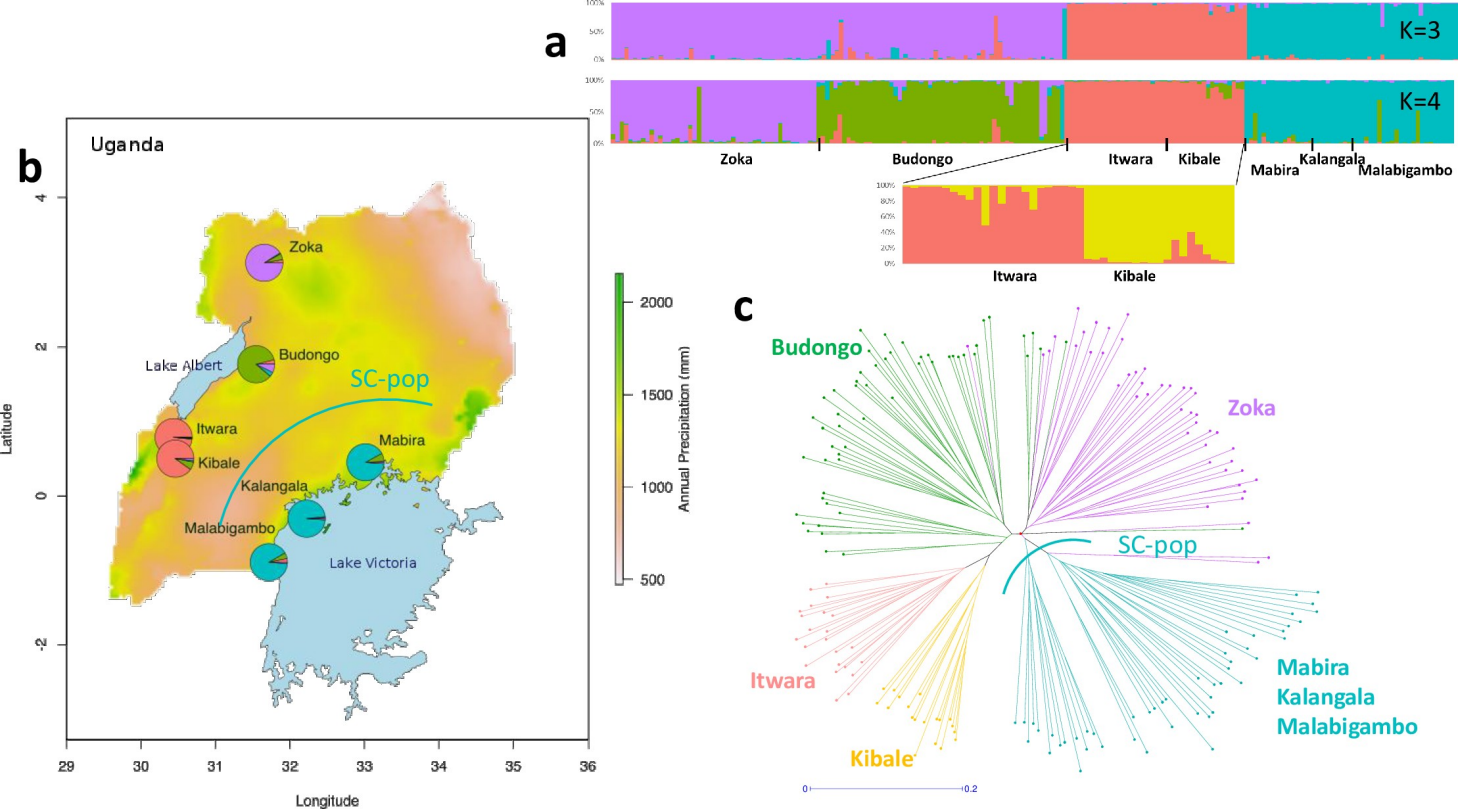
Genetic structure of Ugandan *C*. *canephora* native populations. **a-** Percentage membership of the 191 wild accessions to each of K = 3 or 4 population clusters as inferred by STRUCTURE based on 19 SSR loci. The substructure is presented below for Itwara and Kibale individuals with K = 2 sub-clusters. **b-** Geographical distribution on the Ugandan map with the annual precipitation variable (BIO12) of SSR genetic groups considered according to the STRUCTURE analysis at K = 4. Each location is depicted as a pie chart with the proportional membership of its alleles to each one of the four groups with colours according to graph a. **c-** Unrooted dendrogram produced using the unweighted neighbour-joining method based on genetic dissimilarity among the 191 accessions. The branch colours indicate accessions corresponding to the five clusters from the population structure analysis, as in a-. Individuals from Mabira, Kalangala and Malabigambo forests are intermixed within a same large southern-central population (SC-pop).

Clustering analysis findings for the whole set of native populations clearly revealed that the native diversity broadly differentiated a large group with the accessions from the SC forests (Malabigambo, Mabira and Kalangala) and four forests in northwestern (NW) Uganda (Zoka, Budongo, Itwara and Kibale) (Fig 2A and 2B). In the SC cluster, all individuals were intermixed regardless of their forest of origin. Increasing the number of groups (K) from K = 3 to K = 4, the most likely K values according to [42] (see S2 Fig), separated Zoka and Budongo individuals into two distinct clusters, while an additional Structure analysis of the Itwara-Kibale cluster further distinguished individuals from these two forests (Fig 2A). Neighbour-joining analysis at the individual level exhibited a similar geographically structured distribution of wild accessions (Fig 2C). *C*. *canephora* wild genotypes were generally classified into clades according to their Structure clusters, with a clear separation between individuals from the four NW forests and those from SC forests. Furthermore, individuals from the four different NW forests (Zoka, Budongo, Itwara and Kibale) clustered according with their “forest” counterparts. Few intermixed clusters of individuals from Budongo and Zoka were observed, thus revealing probable gene flow and admixture between adjacent Budongo and Zoka populations. Accessions from SC forests (Malabigambo, Mabira and Kalangala) were spread within a large clade, suggesting population intermixing within the region.

This observed genetic structure was supported by the population divergence (F_*st*_) values (S5 Table). These values were significant for all different forest pairs. Yet, the F_*st*_ values reflected a higher differentiation between the NW populations than between the SC populations. The F_*st*_ values for NW forests ranged from 0.05 (differences between Zoka and Budongo) to 0.18 (differences between Zoka and Itwara), while those for the SC forests, ranged from 0.02 to 0.05. The Fst values indicating the differentiation between each of the four NW forests and other forests, ranged from 0.08 to 0.22. The genetic diversity in Ugandan *C*. *canephora* native populations is thus structured into five main genetic groups: Zoka, Budongo, Itwara and Kibale populations in northwestern Uganda and the SC group in southwestern Uganda.

### Genetic relationships among Ugandan wild, feral, and cultivated accessions

Collections of cultivated material maintained in Kituza and Kawanda, when compared to wild populations, had amongst the highest number of detected alleles, with allelic richness of Rs = 4.7 and 4.78 for material from the Kituza and Kawanda collection, respectively and the highest and equal expected heterozygosity (H_*e*_ = 0.63). However, they presented no or few private alleles (Table 2) that differentiated them from wild populations. SC populations, with very few private alleles, shared most of their alleles with individuals in collections. Conversely, private alleles present in Zoka and Budongo were not represented in the collections.

**Fig 3:**
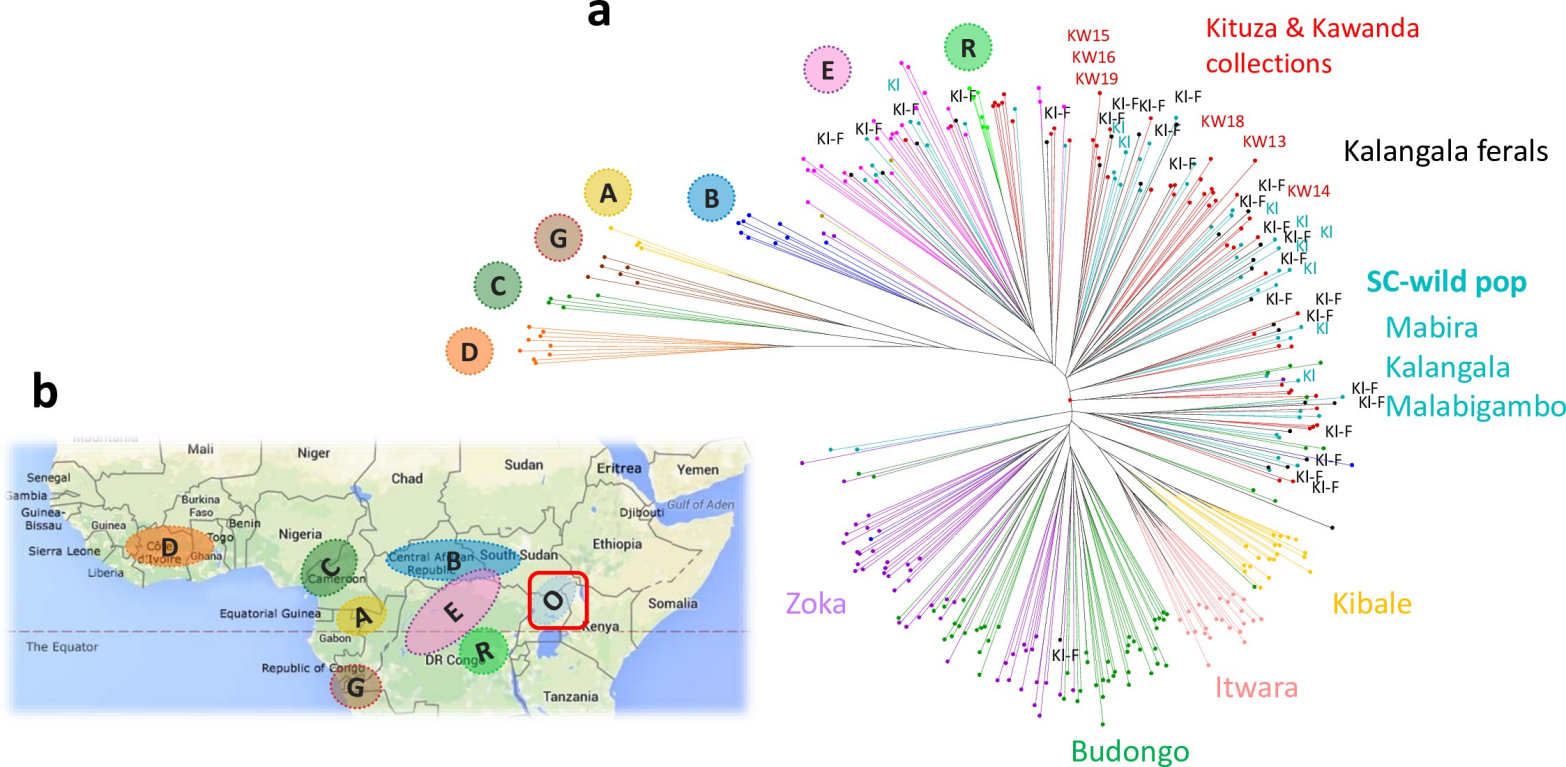
Ugandan *C*. *canephora* diversity relative to the species’ global diversity structure. **a-** Neighbour-joining of Ugandan wild *C*. *canephora* material together with feral accessions from Kalangala islands (noted “Kl-F” in black) and cultivated material maintained in Kituza and Kawanda collections (in red). The six elite clones, KW13, KW14, KW15, KW16, KW18 and KW19 are indicated. Wild material was collected from Zoka, Budongo, Kibale, Itwara, Malabigambo, Mabira and Kalangala islands (Kl). The colours of branches of wild material correspond to the five clusters from the population structure analysis presented in Fig 2. Individuals representative of other genetic groups (A, B, C, D, E, R) from the whole species diversity are also presented for reference**. b**- Eight divergent genetic groups of *C*. *canephora* and their geographical distribution (adapted from [23]).

Neighbor-joining analysis combining cultivated material and feral individuals from Kalangala district islands together with wild ones, showed a closer genetic relationship between the cultivated/feral accessions and the wild ones from the southern-central (SC) forests (Figs 3 and S3). This cluster of SC forest and cultivated accessions tended to be genetically homogeneous, with individuals intermixed irrespective of origin, Nganda or Erecta-derived type (S3 Fig), elite clones or not, and cultivation status. Finally, the cultivated material maintained in the Kituza and Kawanda collections appeared to be highly representative of the diversity found in the SC region, both at the wild and cultivated level, while differing from the other populations (Zoka, Budongo, Itwara and Kibale). Feral individuals, from Kalangala district islands, were also largely scattered within the wild populations from the SC forests (Fig 3). They did not have a closer relationship with their wild counterparts from the Kalangala district islands than with other individuals from the SC region.

### Position of Ugandan accessions among the African C. canephora diversity

When Ugandan *C*. *canephora* genotypes were analyzed together with individuals from seven other diversity groups (A, B, C, D, E, R and G, as described by [23]), they clustered within a distinct genetic group (group O), although a few samples clustered within genetic groups B or E/R (Fig 3A). Indeed, some Ugandan *C*. *canephora* individuals from Zoka clustered with individuals from the Central African Republic (group B), suggesting their close genetic relationship together with their geographic proximity in northern region of Uganda (S3 Fig). In addition, some samples from Mabira and Kalangala forests and from the Kituza and Kawanda collections were grouped together with material from genetic group E or R, suggesting that some cultivated material in Uganda was sourced from the Democratic Republic of the Congo (DRC). Notably, the five distinct genetic structures within native Ugandan *C*. *canephora* diversity were evident amidst other genetic groups (Fig 3).

### Climate characterization of Uganda’s C. canephora native sites

Uganda’s native *C*. *canephora* populations were distributed in forests with significantly varying environmental conditions, as exemplified by five environmental variables including the two independent annual bioclimatic variables (annual mean temperature (°C) -BIO1 and annual precipitation (mm) -BIO12) (Table 1). The *C*. *canephora* populations occurred at different elevations from each other, except Mabira and Kalangala whose elevations did not significantly differ. The Itwara and Zoka populations occurred at the highest and lowest elevations, respectively, with the elevation in Itwara being 562 m.a.s.l higher than that of Zoka. Besides, Zoka had the highest annual mean temperature (BIO1), which was 4.7°C higher than that of the Itwara the population with the lowest BIO1. Similar BIO1 patterns and elevation values were observed across locations since both were correlated (p< 0.05) (S4 Fig). For precipitation, Kalangala had the highest annual precipitation (BIO12), which was 675.5 mm more than that of Zoka, i.e. the location with the lowest precipitation.

Annual precipitation differed significantly across locations, except between Itwara-Malabigambo and Zoka-Kibale (S6 Table). Potential evapotranspiration (PET) was highest in Zoka and lowest in Kalangala. The results showed that PET differed significantly between all sites, except between Itwara and Malabigambo. The aridity index (AI) was highest in Kalangala and was 45.6% higher than the lowest AI observed in Zoka. This implies that, among the study locations, Zoka was the driest and Kalangala the wettest. The AI also differed significantly between locations, except between Budongo and Kibale and between Malabigambo and Itwara.

When all climatic variables were analyzed simultaneously through a principal component analysis (PCA), the populations in Zoka, Budongo, Kalangala, Mabira and Malabigambo occurred in distinct climatic envelopes, while the climatic envelopes in Itwara tended to overlap those of Kibale (Fig 1). In this figure, the first two axes captured ~85% of the total variation in the environmental conditions. The first PCA axis (PC1) accounted for more than half (64.7%) of the total variation and was mainly represented by, and negatively correlated with, temperature-related variables (BIO5, BIO9, BIO4, BIO10, ordered according to their contribution level) and PET. The PC1 axis mainly differentiated populations in warmer areas (Zoka and Budongo) from those in colder areas. The second PCA axis (PC2) accounted for 21.3% of the total variation and was mainly represented by, and negatively correlated with, precipitation-related variables (BIO16, BIO12, BIO13 and Aridity (AI), ordered according to their contribution level). The climatic envelope of Kalangala was characterized by higher precipitation and AI, while the seemingly overlapping climatic envelopes of Itwara and Kibale populations were amongst the driest.

### Relationships between the climatic profiles and the genetic diversity

**Fig 4:**
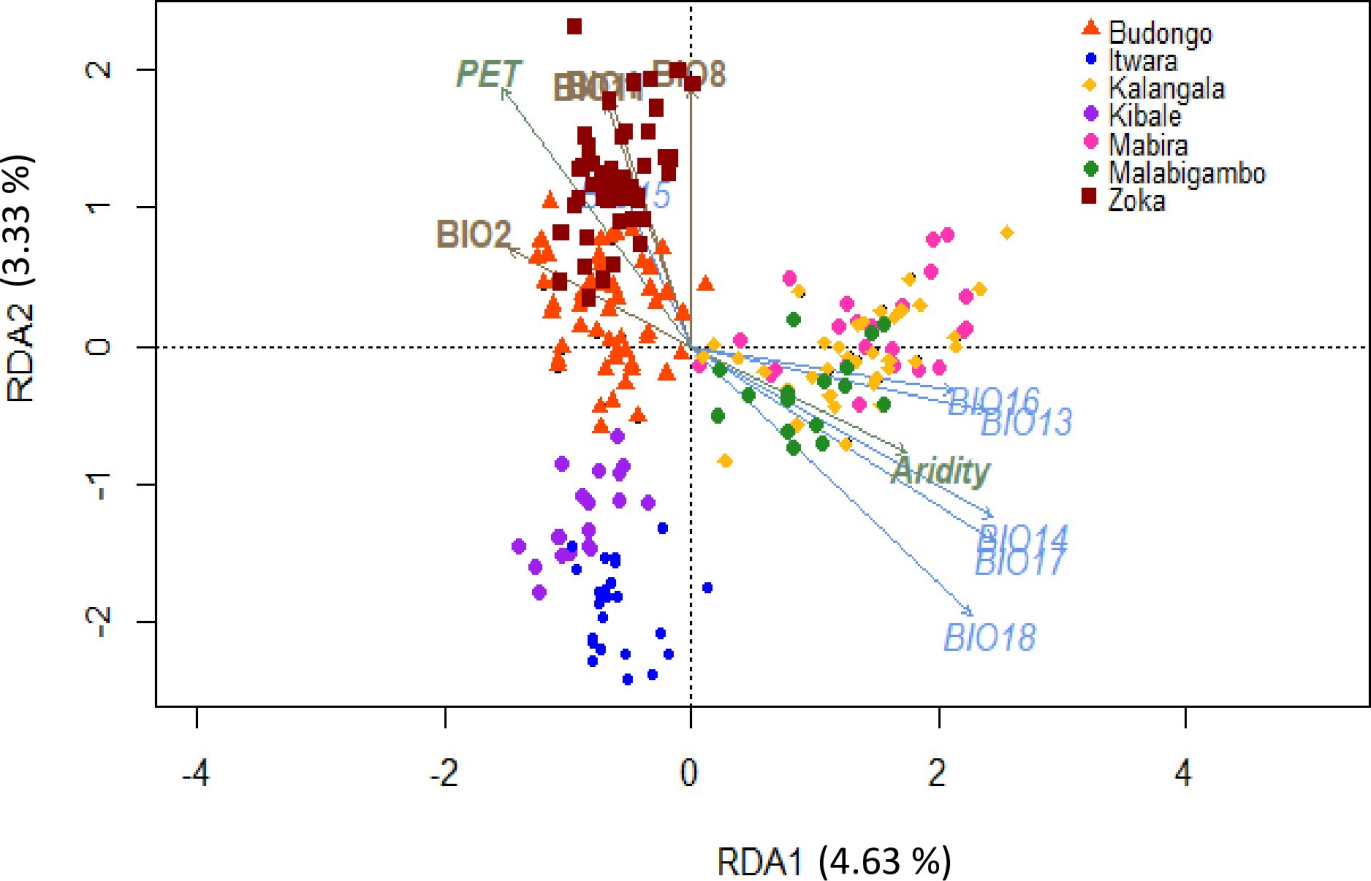
Constrained redundancy analysis (RDA) of the effects of environmental parameters on the allelic diversity of Ugandan *C*. *canephora* wild populations. Twelve out of the 22 environmental variables used in this study significantly and collectively explained 16.3% of the total variation in allelic diversity. Colour codes are the same as in Fig 1. The first and second constraining axes explained 4.63% and 3.33% of the total genetic diversity, respectively.

Comparisons between pairwise individual genetic distances and their geographical distances showed a significant but weak isolation by distance effect (R = 0.347 with *p*<0.001) (S5 Fig). Meanwhile, the constrained redundancy analysis (RDA) results showed that only 12 out of the 22 environmental variables used in this study significantly and collectively explained 16.3% of the total genetic diversity, as defined by the allelic composition (total of 160 alleles) across the study sites (Fig 4). The first constraining axis (RDA1) explained only 4.63% of the total genetic diversity and clearly differentiated the Zoka, Budongo, Kibale and Itwara populations from the Mabira, Malabigambo and Kalangala ones. RDA1 was mainly negatively correlated with the mean diurnal range, *i*.*e*. the mean of monthly (max temperature—min temperature) (BIO2) and PET, while being positively correlated with precipitation-related variables, e.g. precipitation of the driest quarter (BIO17), precipitation in the driest month (BIO14) and precipitation of the wettest month (BIO13). The second axis (RDA2) explained 3.33% of the total genetic diversity and was mainly positively correlated with three temperature-related variables, i.e. mean temperature of the wettest quarter (BIO8), mean temperature of the coldest quarter (BIO11), annual mean temperature (BIO1), and negatively correlated with precipitation in the warmest quarter (BIO18). RDA2 differentiated the genetic diversity of populations occurring in warmer zones (Zoka and Budongo) from others. Interestingly, the RDA projection structured individuals according to their forest of origin for northwestern (NW) populations (Zoka, Budongo, Kibale, Itwara), but individuals from southern-central (SC) populations (Kalangala, Mabira, and Malabigambo) all overlapped in the two first axis spaces. However, the low proportion of constrained variance, i.e. 16.3% of the total genetic diversity, suggested that there are also other factors affecting genetic diversity and structure of *C*. *canephora* in the region.

## Discussion

### Genetic differentiation of Ugandan wild C. canephora populations

Our results showed that the genetic diversity found within Ugandan wild *C*. *canephora* populations was not present elsewhere in the *C*. *canephora* distribution range. The studied material constituted a well-differentiated genetic group, as previously suggested by [23] who studied a small sub-set of three of our individuals. This diversity was broadly structured in five differentiated genetic groups, namely four groups in northwestern (NW) Uganda, including individuals from Zoka, Budongo, Kibale and Itwara forests, and a southern-central (SC) group containing accessions from Malabigambo, Mabira and Kalangala wild populations.

Forest populations from the SC cluster had higher allelic richness than those from the NW forests, except Zoka forest. They however constituted a large intermixed genetic group, which could possibly be attributed to a high level of gene flow between nearby forest and plantations. The relatively low allelic diversity that we found for the western Ugandan material might be due to restricted gene-flow between nearby populations isolation by fragmentation, genetic drift and bottleneck caused by anthropogenic disturbances in these forests, or selective mortality under local stress event. This factors could potentially also explain the significant genetic divergence noted for Itwara and Kibale populations(. Indeed, there are indications that Itwara and Kibale were once part of a larger connected continuum but in the early 1900s areas in between were allocated to human settlement, and the two forests are now 30 km apart [31,52]. Musoli et al. [31] and Nyakaana [53] also found that populations from Itwara and Kibale were genetically distinct, with low allelic diversity, possibly indicating inbreeding combined with random genetic drift. Moreover, our sample collection surveys revealed a marked reduction in the spatial range occupied by the *C*. *canephora* populations in these forests compared to that previously described by [31]. Among all forest populations studied, Zoka and Budongo populations were highly differentiated and showed the highest level of originality in terms of number of private alleles per locus. This indicates that these populations include so far unexplored genetic material, which could be of great potential interest to the coffee breeding sector.

### Gene flow between wild southern-central populations and cultivated Ugandan coffees

Most cultivated germplasm of Uganda seem to have originated from the southern-central forests (Malabigambo, Kalangala, Mabira) (see S1 Table). There is also evidence of the introduction of cultivated genotypes from Congo/DRC (genetic group E/R, S3 Fig). The genetic similarity between the cultivated samples and the wild material from SC forests is consistent with the fact that, in the late 19^th^ century, smallholder farmers in the Lake Victoria Basin region began cultivating *C*. *canephora*, probably using directly sourced wild coffee material [3,35,52]. The location of the Mabira, Malabigambo and Kalangala forests coincides with the predominant and historically important *C*. *canephora* cultivation in these zones of Uganda. Moreover, the pollen (*via* wind or bees) and seed dispersal (*via* primates or bats) of the species [17,18] might have increased forest connectivity, especially for those surrounded by coffee plantations. Thus, the genetic similarity between the SC wild samples and the cultivated material could reflect both their close genetic origin and the gene flow between forest material and the plantations, hence explaining the lack of clear delineation between the SC wild and cultivated samples. Moreover, human interaction, in the form of gathering and transporting wild *C*. *canephora* plants from SC forests for cultivation use, led to the spread of seeds and/or cuttings over large distances within the *C*. *canephora* cultivation areas of southern-central Uganda [35]. Meanwhile, some cultivated genotypes clustered with Congolese genetic group E/R, which very likely reflects the introduction of Congolese germplasm in Uganda. In particular, it has been suggested that the Nganda type may have originated from wild coffee sampled from local forest, whereas the Erecta type was introduced from the Congo Basin [35]. However, they appeared as intermixed with individuals of the SC group, both from E/R and local origin, meaning that their phenotypic differences were not clearly distinguishable by the genetic markers (S4 Fig). Indeed, the two forms are often grown in mixtures and freely cross [35]. This corroborates the suggestion of [54] that some cultivated coffee trees in Uganda might have resulted from natural crosses between wild endemic materials and introduced genotypes, thus giving rise to mixed genotypes.

The relative contributions of the aforementioned factors, i.e. (i) use of wild material by farmers, (ii) use of introduced material from abroad, and (iii) natural gene flow between populations are hard to assess. However, when we compare the genetic diversity indices between the SC wild accessions (He = 0.62) with wild accessions from different regions of Africa pooled together (He = 0.61 according to [22]), they are similar. The lack of genetic differences between cultivated, local or introduced, and wild material in the SC region may thus reflect that the natural or unique diversity in native *C*. *canephora* populations is well-distributed in both wild and cultivated accessions.

### Feral and wild accessions from Kalangala

We expected that wild samples from Kalangala would show a substantial level of originality due to the genetic material collected from the Lutooboka central forest reserve [52]. However, our results revealed that material from Kalangala was not genetically distinct from that collected in other SC forests and in the cultivated collections. This lack of differentiation could have resulted from increased deforestation, hence reducing the extent of wild coffee populations in Kalangala. Most natural coffee populations in Kalangala have been cleared to make way for coffee or oil palm plantations [55]. Consequently, our wild accessions from the highly fragmented natural forests of Kalangala could have been cultivated *C*. *canephora* offspring or accessions introgressed with cultivated material. Indeed, local feral individuals, formerly cultivated material returned to the wild, were related to the individuals from the SC group. We found a much greater genetic similarity between the wild and cultivated accessions than Musoli et al. [31]. This might be due to the reasons already mentioned before, i.e., the high gene flow rates and the origin of the cultivated accessions, since most of them are from the Lake Victoria region (see S1 Table). The genetic diversity indices were still high in this wild Kalangala population if considering the threat to the wild populations there and the small sample size (n = 10) of wild material from this location in our study.

### Distinct climatic zones of Ugandan C. canephora native populations

Our study showed that Ugandan *C*. *canephora* populations occurred across a broad environmental range, except for Kibale and Itwara whose climatic envelops tended to overlap (Fig 1). These findings were in line with our expectations because Ugandan *C*. *canephora* distribution range occurs in five climatic zones with different rainfall levels [32,34]. The Zoka population was found in the driest climatic envelope, with high temperatures and comparatively highly fluctuating rainfall, while the Kalangala population occupied the wettest climatic envelope where high rainfall and comparatively low temperature fluctuations prevailed (Fig 1 and Table 1). Our results showed that Ugandan natural *C*. *canephora* populations occurred in contrasting environmental conditions, which makes it possible that these populations became adapted to these conditions. Intraspecific variability of plant populations has been reported to enable species to thrive under new environmental conditions [56–59]. Such variation within and across populations is important as it could provide essential material for addressing abiotic and biotic challenges in the coffee sector. However, the low proportion of constrained variance of the environmental variables indicated that there were other factors *e*.*g*. gene flow and genetic drift or bottlenecks that probably result from deforestation and fragmentation, affecting genetic diversity and structure of Uganda’s wild *C*. *canephora* populations. In particular, while there was a clear environmental variation among SC regions (Fig 1A), the SC populations were intermixed within the same genetic group and overlap each other in the constraint genetic variance analysis of the environmental variables (Fig 4). Thus, in spite of the environmental variation, factors such as above mentioned gene flow and material mixing most have strongly affected the SC populations.

### Utilization of Ugandan coffee genetic diversity and the need for a complementary conservation strategy

Ugandan *C*. *canephora* production is currently predominantly sustained by six elite clones, namely: KW13, KW14, KW15, KW16, KW18 and KW19. These are specially bred for their resistance to coffee wilt disease (CWD) and high productivity. In this study, all six cultivars were shown to be genetically similar to SC wild populations. Our results showed that the genetic diversity in NW forests was distinct from that of the cultivated material and that NW populations occurred in comparatively contrasted climatic zones, thus highlighting a new source of genetic diversity. This unexplored genetic diversity could be utilized in coffee breeding programs to improve the resilience of cultivated material to various adverse effects of climate change, e.g. drought, temperature, pests and diseases.

In the light of the substantial level of Ugandan *C*. *canephora* diversity and genetic structure and the location of the populations in distinct environmental habitats, there is need to devise an efficient complementary conservation strategy that allows for the *in-situ* and *ex-situ* conservation of Ugandan coffee genetic resources. As shown earlier, some populations like those of Itwara and Kibale are small spatially isolated populations which could thus be more susceptible to ecological or genetic disturbance [60]. In our study, accessions from Zoka forest, and to a lesser extent Budongo forest, were found to contain the highest number of mean private alleles per locus, which could possibly be explained by the lower human disturbance in these areas, the higher level of isolation, etc. As these forests lie in areas with the most arid environmental conditions, these populations might also contain some unique genetic diversity with adaptive potential to local environment. Hence both *in-situ* and *ex-situ* conservation of these populations is especially important. Conservation strategies should promote the prevention of deforestation and associated habitat destruction in these areas. For *ex-situ* conservation, our study enriched the diversity conserved by the National Agricultural Research Organization (NARO) at the National Coffee Resources Institute (NaCORI) in Kituza and the Plant Genetic Resources Centre (PGRC) in Entebbe. Our study material was safety duplicated to enhance the *ex-situ* conservation of these resources and provide material for other related studies geared towards understanding the potential offered by Ugandan coffee genetic diversity.

In conclusion, our study revealed that Ugandan *C*. *canephora* populations thrived in contrasting environmental conditions and that the genetic structuring of wild populations was divided into five main environmentally and geographically bounded groups, i.e. the four northwestern forests (Zoka, Budongo, Itwara and Kibale) and the southern-central group, mainly comprising wild populations from Malabigambo, Mabira and Kalangala, and the cultivated collection material. We also demonstrated that the distinction between populations was partly correlated with the climatic differences in their habitats, suggesting that these populations might present an adaptive potential useful for breeding climate change resilient material. Most importantly, our study showed that the cultivated and current elite genotypes predominantly belonged to the same genetic group (SC group), while material from the NW group (especially from Zoka and Budongo) contained genetic material that has not yet been utilized but which could be very useful for coffee improvement in the current climate change setting. *Ex-situ* conservation strategies must be developed very quickly and the material already collected in the national collections should be evaluated in more detail, in particular for its physiological capacities to cope with the adverse effects of climate change, e.g. drought, higher temperatures or disease pressure [61].

## Supporting information

S1 Fig Eight divergent genetic groups of *C*. *canephora* and their geographical distribution (adapted [23]). (PDF)

S2 Fig Optimal number of genetic clusters in the wild Ugandan *C*. *canephora* set. (PDF)

S3 Fig Neighbour-joining dendrogram of Ugandan wild *C*. *canephora* material together with cultivated material maintained in Kituza and Kawanda collections. (PDF)

S4 Fig Pearson correlation coefficients between 22 environmental variables. (PDF)

S5 Fig Isolation by distance (IBD). (PDF)

S6 Fig Neighbour-joining dendrogram of Ugandan wild *C*. *canephora* material from the souther-center (SC) population (Mabira, Kalangala and Malabigambo forests) together with cultivated material maintained in Kituza and Kawanda collections and ferals. (PDF)

S1 Table Genetic and climatic raw data, cultivated material description. (XLSX)

S2 Table Representative individuals from the *C*. *canephora* genetic groups defined by [21] and their origins. (PDF)

S3 Table Description of the 19 SSR markers used for DNA fingerprinting of the Ugandan coffee samples, including their forward and reverse primer sequences. (PDF)

S4 Table Environmental variables. (PDF)

S5 Table Differentiation (F_*st*_) between native sites. (PDF)

S6 Table Pairwise *t*-test results to test differences between locations across locations and variables. (PDF)
