# Supplementary material for: Genetic diversity of native and cultivated Ugandan Robusta coffee (Coffea canephora Pierre ex A. Froehner): Climate influences, breeding potential and diversity conservation
Source: PLoS One. 2021 Feb 8;16(2):e0245965. doi: 10.1371/journal.pone.0245965 (PMC7870046; doi:10.1371/journal.pone.0245965)
Supplement: S1 Fig — (PDF) [file pone.0245965.s001.pdf]

Supplementary figure S1. Eight divergent genetic groups of *C. canephora* and their geographical distribution (adapted from Merot-L'anthoene et al., 2019): **A-** Geographical distribution of eight genetic groups, **B-** A neighbor-joining tree based on Euclidean distance between the eight a priori groups of *C. canephora* and accessions of *C. arabica* and *C. eugenioides,* **C-** Summary table of the historical definition of *C. canephora* genetic groups with the references and the marker types in use. Ugandan group is highlighted in red.

a b

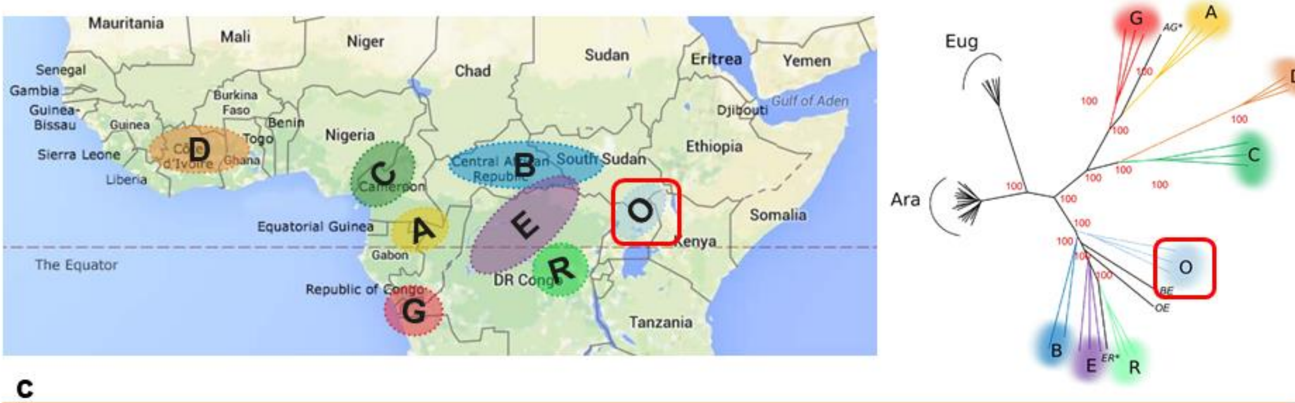

c

Study	Berthaud, 1986	Montagnon et al., 1992	Cubry et al., 2008<br>Musoli et al. 2009	Dussert et al., 1999<br>Gomez et al., 2009	Mérot-L'Anthoene et al. 2019	Geographic origin
Type	Isozymes	Isozymes	SSR	RFLP, SSR	SNP	Wild
Genetic groups	Guinean	Guinean	Guinean	D	D	Guinea, Côte d'Ivoire
	Congolese	SG1	SG1	A	A	North of Congo, South of Cameroon
		SG2	SG2	B	B	East of Central Afr. Rep.
				E	E	Democr. Rep of the Congo, Cameroon
		Not included	C	C	C	West of Central Afr. Rep., Cameroon
	Not included		Ug	Not included	O	Uganda
			Not included	Not included	R	South of the Democr. Rep of the Congo
					G	North and West of Angola

Berthaud J. Les ressources génétiques pour l'amélioration des caféiers africains diploïdes. Collection Travaux et documents. Montpellier, France: Orstom; 1986.

Montagnon C, Leroy T, Yapo A. Genotypic and phenotypic diversity of some coffee groups (*Coffea Canephora* Pierre) in the collections - consequences on their use in breeding. Cafe Cacao The. 1992;36: 187–198.

Cubry P, Musoli P, Legnate H, Pot D, de Bellis F, Poncet V, et al. Diversity in coffee assessed with SSR markers: structure of the genus *Coffea* and perspectives for breeding. Genome. 2008;51. doi:10.1139/g07-096

Musoli P, Cubry P, Aluka P, Billot C, Dufour M, De Bellis F, et al. Genetic differentiation of wild and cultivated populations: diversity of *Coffea canephora* Pierre in Uganda. Genome. 2009;52: 634–46.

Dussert S, Lashermes P, Anthony F, Montagnon C, Trouslot P, Combes MC, et al. Coffee, *Coffea canephora*. In: Hamon P, Seguin M, Perrier X, Glaszmann J-C, editors. Diversité génétique des plantes tropicales cultivées. Montpellier, France: CIRAD; 1999. pp. 175–794.

Gomez C, Dussert S, Hamon P, Hamon S, de Kochko A, Poncet V. Current genetic differentiation of *Coffea canephora* Pierre ex A. Froehn in the Guineo-Congolian African zone: cumulative impact of ancient climatic changes and recent human activities. BMC evolutionary biology. 2009;9: 167. doi:10.1186/1471-2148-9-167

Mérot-L'Anthoene V, Tournebize R, Darracq O, Rattina V, Lepelley M, Bellanger L, et al. Development and evaluation of a genome-wide Coffee 8.5K SNP array and its application for high-density genetic mapping and for investigating the origin of *Coffea arabica* L. Plant biotechnology journal. 2019. doi:10.1111/pbi.13066
