# Supplementary material for: Genetic diversity of native and cultivated Ugandan Robusta coffee (Coffea canephora Pierre ex A. Froehner): Climate influences, breeding potential and diversity conservation
Source: PLoS One. 2021 Feb 8;16(2):e0245965. doi: 10.1371/journal.pone.0245965 (PMC7870046; doi:10.1371/journal.pone.0245965)

**Supplementary Figure S2**. Optimal number of genetic clusters in the wild Ugandan *C. canephora* set according to the admixture model with correlated alleles implemented in Structure. a The log-likelihood of the data [lnP(X)] averaged over 10 consecutive Structure runs for K = 2 to 7, with error bars representing ± standard deviation. b Evanno's ΔK statistic plotted against K.

Structure analysis revealed a maximum ΔK value for K = 3 suggesting the split into three main clusters, but it is worth noting that for K = 4, ΔK value is above zero indicating that the genetic structure produced by dividing all genotypes into 4 groups is also likely.

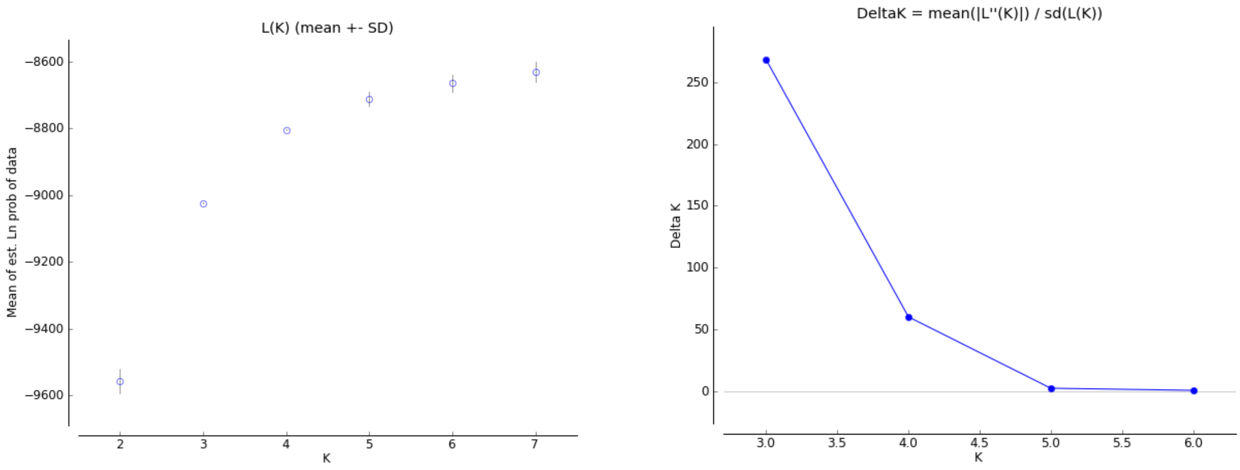

Supplement: S2 Fig — (PDF) [file pone.0245965.s002.pdf]
