# Supplementary material for: Genetic diversity of native and cultivated Ugandan Robusta coffee (Coffea canephora Pierre ex A. Froehner): Climate influences, breeding potential and diversity conservation
Source: PLoS One. 2021 Feb 8;16(2):e0245965. doi: 10.1371/journal.pone.0245965 (PMC7870046; doi:10.1371/journal.pone.0245965)

Supplementary figure S3. Neighbour-joining dendrogram of Ugandan wild *C. canephora* material together with cultivated material maintained in Kituza and Kawanda collections (in red). The Erecta- and Nganda- derived varieties are indicated. Wild material was structured in 5 genetic groups: Zoka, Budongo, Kibale, Itwara, and southercenter (SC). Individuals representative of other genetic groups (B, E, R) from the whole species diversity are also presented for reference. Samples from SC-pop and from the Kituza and Kawanda collections were grouped together with material from genetic group E or R, suggesting that some cultivated material in Uganda was sourced from the Democratic Republic of the Congo (DRC). Some individuals from Zoka clustered with individuals from the Central African Republic (group B), suggesting their close genetic relationship together with their geographic proximity in northern region of Uganda.

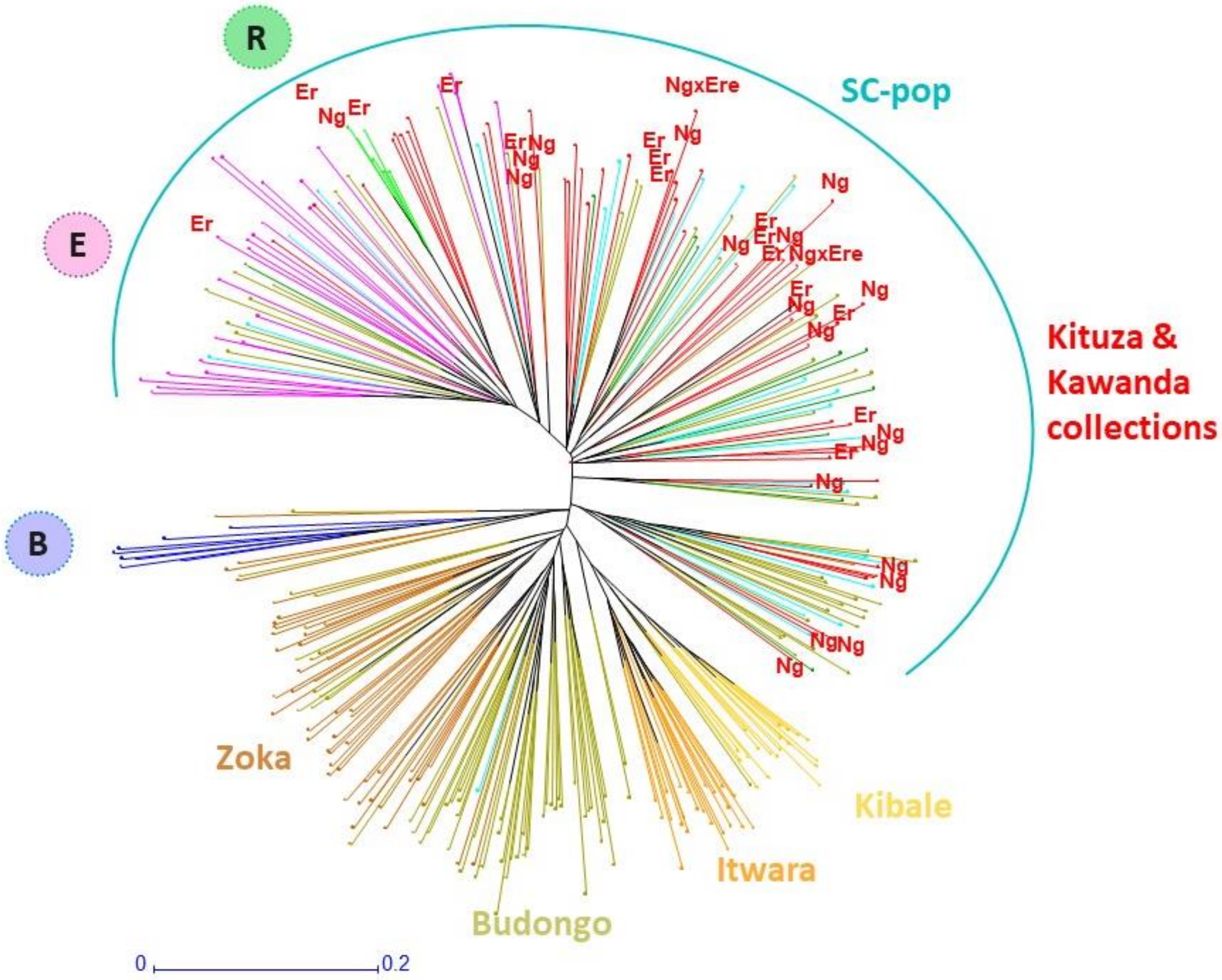

Supplement: S3 Fig — (PDF) [file pone.0245965.s003.pdf]
