# Supplementary material for: Genetic diversity of native and cultivated Ugandan Robusta coffee (Coffea canephora Pierre ex A. Froehner): Climate influences, breeding potential and diversity conservation
Source: PLoS One. 2021 Feb 8;16(2):e0245965. doi: 10.1371/journal.pone.0245965 (PMC7870046; doi:10.1371/journal.pone.0245965)

Supplementary Figure S4 Pearson correlation coefficients between 22 environmental variables. The blue and red colors indicate positive and negative correlations, respectively, while the color intensity and circle size are proportional to the correlation coefficients. The right side of the figure shows the legend displaying the correlation coefficients and the corresponding colors significant at $p \leq 0.05$

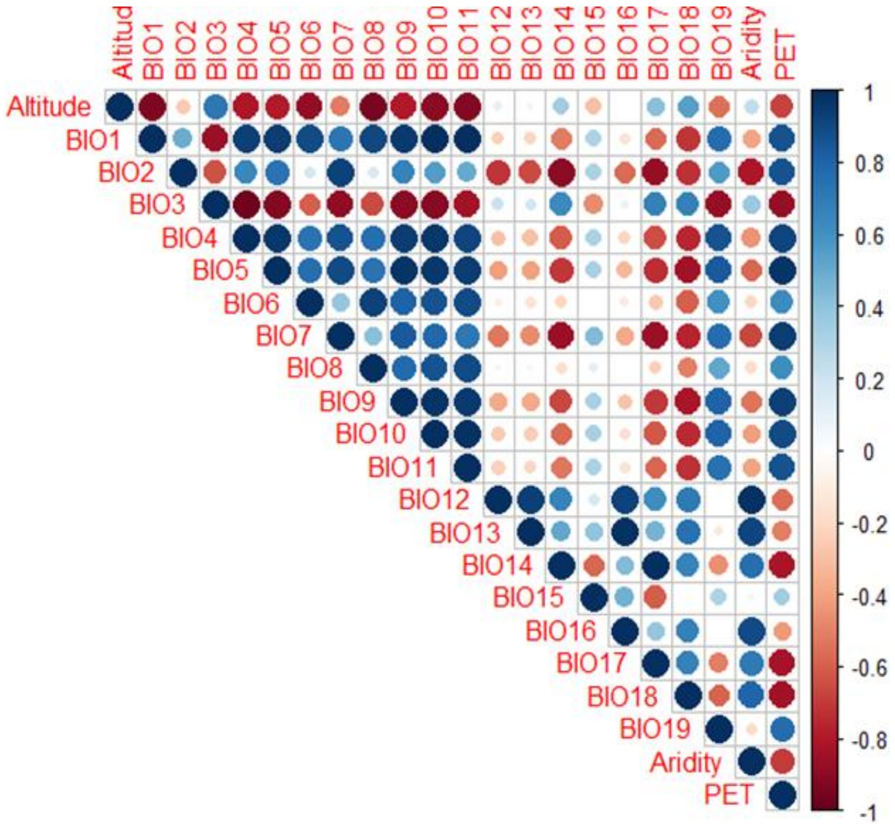

Supplement: S4 Fig — (PDF) [file pone.0245965.s004.pdf]
