# Supplementary material for: Genetic diversity of native and cultivated Ugandan Robusta coffee (Coffea canephora Pierre ex A. Froehner): Climate influences, breeding potential and diversity conservation
Source: PLoS One. 2021 Feb 8;16(2):e0245965. doi: 10.1371/journal.pone.0245965 (PMC7870046; doi:10.1371/journal.pone.0245965)

Supplementary Figure S5. Isolation by distance (IBD): Correlation between the pairwise genetic distances of Ugandan *C. canephora* wild individuals with pairwise geographical distances (Mantel test with 999 permutations at p=0.01).

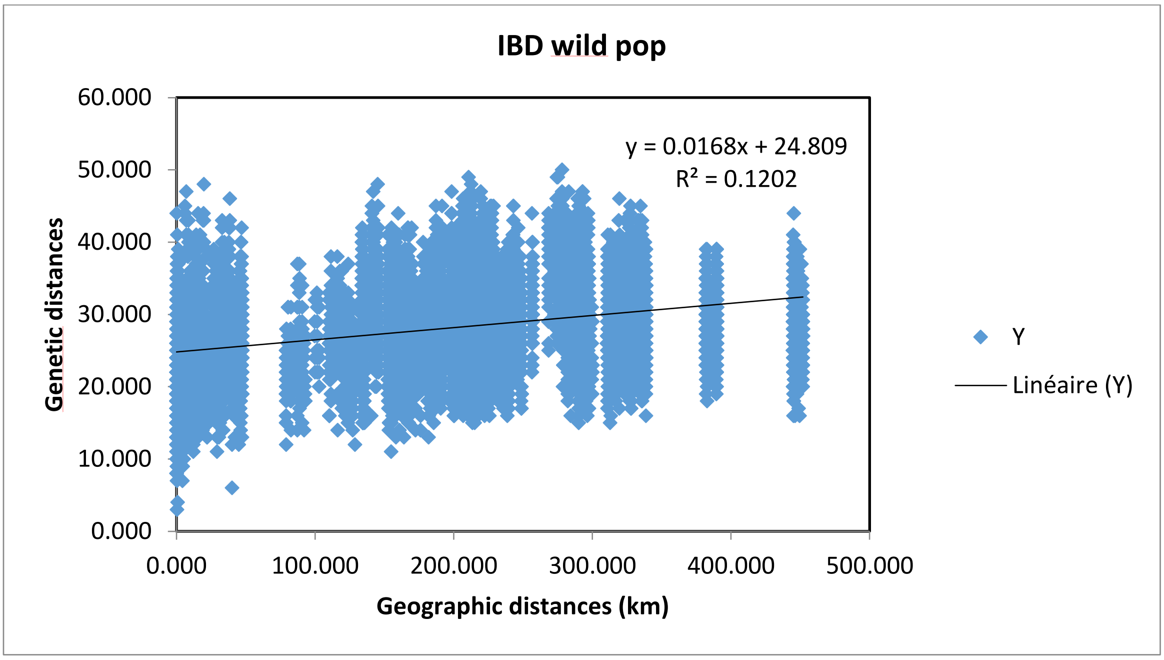

Supplement: S5 Fig — (PDF) [file pone.0245965.s005.pdf]
