# Supplementary material for: Genetic diversity of native and cultivated Ugandan Robusta coffee (Coffea canephora Pierre ex A. Froehner): Climate influences, breeding potential and diversity conservation
Source: PLoS One. 2021 Feb 8;16(2):e0245965. doi: 10.1371/journal.pone.0245965 (PMC7870046; doi:10.1371/journal.pone.0245965)
Supplement: S2 Table — (PDF) [file pone.0245965.s008.pdf]

Supplementary Table S2. Representative individuals from the *C. canephora* genetic groups defined by Mérot-L'Anthoene et al. (2019) and their origins. Group O individuals are in common with the present study: MB3.1, ML5.1 and ZK2.5.

Genetic group	No	Status	Country	Collection	No
A	4	Wild	Congo	CNRA	3
			Gabon	Meise	1
B	8	Wild	Central African Republic	CNRA	3
			"	IRD	3
			Democratic Republic of the Congo	Meise	2
C	6	Wild	Cameroon	CNRA	4
			Congo	CNRA	1
			Central African Republic	CNRA	1
D	9	Wild	Côte d'Ivoire	CNRA	8
			Republic of Guinea	CNRA	1
E	23	Wild	Cameroon	Meise	1
			"	CNRA	4
			Congo	CNRA	7
			Côte d'Ivoire	CNRA	3
			Central African Republic	CNRA	2
			Democratic Republic of the Congo	Meise	2
				ICCRI	4
G	6	Wild	Angola	INCA	6
R	6	Wild	Democratic Republic of the Congo	ICCRI	6
O	3	Wild	Uganda	NARO	3
Total	65				

CNRA: Centre national de recherche agronomique, Côte d'Ivoire ; Meise : Meise Botanic Garden, Belgium ; INCA: Instituto Nacional do Café, Angola ; ICCRI: Indonesian Coffee and Cocoa Research Institute, Indonesia ; NARO: National Agricultural Research Organization.
