# Supplementary material for: Genetic diversity of native and cultivated Ugandan Robusta coffee (Coffea canephora Pierre ex A. Froehner): Climate influences, breeding potential and diversity conservation
Source: PLoS One. 2021 Feb 8;16(2):e0245965. doi: 10.1371/journal.pone.0245965 (PMC7870046; doi:10.1371/journal.pone.0245965)
Supplement: S3 Table — (PDF) [file pone.0245965.s009.pdf]

Supplementary Table S3. Description of the 19 SSR markers used for DNA fingerprinting of the Ugandan coffee samples, including their forward and reverse primer sequences, repeat motif, annealing temperature (Tm), expected amplicon size and multiplex set. The linkage group (LG) and chromosome (Chr.) of *C. canephora* belonging are indicated (except nd: not determined). Complementary information can be found in MoccaDB (http://moccadb.ird.fr/) and in the coffee genome hub (http://coffee-genome.org/).

Marker	Primer code	Forward Primer	Reverse Primer	Repeat motif	Tm (°C)	Size (bp)	Multiplex set	MoccaDB code	LG	Chr.
R338	R338	CGAAGGCTGTCAACAACTGG	GGGATAAACAAGTTAAAGGA	$(GA)_{17}$	60	241	1	nd	E	5
SSR146	122850	TCCAGTTTGATCAGCAACCA	CCATCTTGGGGATAGAGCAA	$(GA)_{16}$	60	153	1	nd	F	6
R278	R278	TGTAGATTTGAAACCCAATC	AAGTCTCGACAAGTTTTGAC	$(GA)_{16}$	60	143	1	R278	E	5
R339	R339	ATTATGCTCGCTGGGCTGTT	TGGGATCACTCCTGTGTCGC	$(CT)_{9}$	60	218	1	R339	G	7
R336	R336	TTGCCTTTTTAGTGCGTGTA	GCAAAGCCCGAGGATT	$(GA)_{4}$	60	177	1	nd	A	1
R325	R325	CCTTGTTGTTGGGGAATGTC	GGCTGTTCTGGGCTTTGTG	$(GA)_{23}$	60	254	2	R325	F	6
SSR196	124195	ATCCCCATCAGAAGACCTCA	CCTCCACCGCCTGTTTATTA	$(GCCA)_{6}$	60	88	2	nd	C	3
R301	R301	CTCACCCCAGCATTTAGAGT	GTTATTTGCCCCATCAGG	$(GA)_{15}$	60	257	2	R301	D	4
R268	R268	GTATCCCACAATGAAATCAC	AGTAGAATTTTCAACATATAAG	$(GA)_{19}$	60	155	2	R268	G	7
R168	R168	CCTGGACTGGTAGAAACAAA	AAAGGTGTTCAATGCCTACA	$(GA)_{18}$	60	265	2	R168	A	1
R189	R189	GGAGTGAGAGGAGGGCGTAG	GAGAGAGGGACACTGCTGC	$(GCA)_{9}(GA)_{15}$	60	235	3	R189	F	6
SSR495	124161	TCGGCTCCCAAATATTCATC	CATGAGGCAAGAGGGTTTGT	$(GA)_{5}$	60	164	3	ssr124161	A	1
SSR497	123909	AGGCTTGCTGGAACTCTTGA	GAAAGACTTGTCCTTTGCCG	$(GA)_{14}$	60	266	3	ssr123909	B	2
R175	R175	GCAGTGACGCAGCAATG	AAAAGGAGAGCCAAAGCAGT	$(GA)_{20}$	60	234	3	R175	F	6
R250	R250	GCATCATTGGGTTGGTGG	CGACTTTCCGCACGCAAAC	$(GA)_{17}$	60	227	4	R250	I	9
R148	R148	CGTCGTTCGAGGACTTGTTC	TTCGCAATCCCAGACCC	$(GA)_{10}(GAT)_{8}$	60	198	4	R148	D	4
SSR209	119699	GCCGTGGTGGAAGATGTACT	CGAGTTCACCAAGAACGTCA	$(AT)_{5}$	60	100	4	ssr119699	A	1
R342	R342	GCGAGAATAAGGAGTGACC	GTCCCTTTTTGTCTGGACC	$(GA)_{9}$	60	151	4	nd	G	7
SSR533	123557	ATCTCCTCGTTCTTCCCCAT	GCTTGTAGCAGGCAGGAAAC	$(GA)_{8}$	60	233	4	ssr123557	B	2
