# Supplementary material for: Genetic diversity of native and cultivated Ugandan Robusta coffee (Coffea canephora Pierre ex A. Froehner): Climate influences, breeding potential and diversity conservation
Source: PLoS One. 2021 Feb 8;16(2):e0245965. doi: 10.1371/journal.pone.0245965 (PMC7870046; doi:10.1371/journal.pone.0245965)
Supplement: S4 Table — (PDF) [file pone.0245965.s010.pdf]

Supplementary Table S4. Environmental variables. The 19 bioclimatic (BIOx) variables averaged for the years 1950-2000 were downloaded from WorldClim database (www.worldclim.org) at 30 arc-second resolution. Elevation was determined in the field by GPS (Garmin eTrex 10 Navigation device). The aridity index (AI) and potential evapotranspiration (PET) data were sourced from the Global-Aridity dataset.

**Environmental variables**	***Abbreviation***
Annual mean temperature (°C)	BIO1
Mean diurnal range (°C)	BIO2
Isothermality (BIO2/BIO7) (* 100) (°C)	BIO3
Temperature seasonality (°C)	BIO4
Max temperature of the warmest month (°C)	BIO5
Min temperature of the coldest month (°C)	BIO6
Temperature annual range (BIO5-BIO6) (°C)	BIO7
Mean temperature of the wettest quarter (°C)	BIO8
Mean temperature of the driest quarter (°C)	BIO9
Mean temperature of the warmest quarter (°C)	BIO10
Mean temperature of the coldest quarter (°C)	BIO11
Annual precipitation (mm)	BIO12
Precipitation of the wettest month (mm)	BIO13
Precipitation of the driest month (mm)	BIO14
Precipitation seasonality (mm)	BIO15
Precipitation of the wettest quarter (mm)	BIO16
Precipitation of the driest quarter (mm)	BIO17
Precipitation of the warmest quarter (mm)	BIO18
Precipitation of the coldest quarter (mm)	BIO19
Elevation (m.a.s.l.: meter above sea level)	ALT
Aridity index	AI
Potential evapotranspiration	PET
