# Supplementary material for: Genetic diversity of native and cultivated Ugandan Robusta coffee (Coffea canephora Pierre ex A. Froehner): Climate influences, breeding potential and diversity conservation
Source: PLoS One. 2021 Feb 8;16(2):e0245965. doi: 10.1371/journal.pone.0245965 (PMC7870046; doi:10.1371/journal.pone.0245965)
Supplement: S5 Table — (PDF) [file pone.0245965.s011.pdf]

Supplementary Table S5. Differentiation (F_{st}) between native sites**.** F_{st} values are below the diagonal, and probabilities, *i.e.* P(rand ≥ data) based on 999 permutations, are shown above the diagonal.

		**Southern-central (SC)-forests**			**North-western (NW)- forests**			
		**Kalangala**	**Mabira**	**Malabigambo**	**Kibale**	**Itwara**	**Budongo**	**Zoka**
**SC-forests**	**Kalangala**		0.045	0.011	0.001	0.001	0.001	0.001
	**Mabira**	0.018		0.001	0.001	0.001	0.001	0.001
	**Malabigambo**	0.036	0.053		0.001	0.001	0.001	0.001
**NW- forests**	**Kibale**	0.216	0.205	0.170		0.001	0.001	0.001
	**Itwara**	0.216	0.176	0.145	0.150		0.001	0.001
	**Budongo**	0.115	0.119	0.084	0.127	0.152		0.001
	**Zoka**	0.112	0.099	0.095	0.149	0.180	0.055	
