# Supplementary material for: Genetic diversity of native and cultivated Ugandan Robusta coffee (Coffea canephora Pierre ex A. Froehner): Climate influences, breeding potential and diversity conservation
Source: PLoS One. 2021 Feb 8;16(2):e0245965. doi: 10.1371/journal.pone.0245965 (PMC7870046; doi:10.1371/journal.pone.0245965)
Supplement: S6 Table — (PDF) [file pone.0245965.s012.pdf]

Supplementary Table S6 Pairwise *t*-test results to test differences between locations across locations of variables

Elevation (m.a.s.l.)

	Budongo	Itwara	Kalangala	Kibale	Mabira	Malabigambo
Itwara	0.000					
Kalangala	0.000	0.000				
Kibale	0.000	0.000	0.000			
Mabira	0.000	0.000	***0.480***	0.000		
Malabigambo	0.000	0.000	0.000	0.000	0.000	
Zoka	0.000	0.000	0.000	0.000	0.000	0.000

Annual mean temperature (°C) - BIO1

	Budongo	Itwara	Kalangala	Kibale	Mabira	Malabigambo
Itwara	0.000					
Kalangala	0.000	0.000				
Kibale	0.000	0.000	0.000			
Mabira	0.000	0.000	0.000	0.000		
Malabigambo	0.000	0.000	0.000	0.000	0.000	
Zoka	0.000	0.000	0.000	0.000	0.000	0.000

Annual precipitation (mm) - BIO12

	Budongo	Itwara	Kalangala	Kibale	Mabira	Malabigambo
Itwara	0.000					
Kalangala	0.000	0.000				
Kibale	0.003	0.000	0.000			
Mabira	0.000	0.000	0.000	0.000		
Malabigambo	0.000	0.786	0.000	0.000	0.013	
Zoka	0.000	0.000	0.000	0.957	0.000	0.000

Potential evapotranspiration (PET)

	Budongo	Itwara	Kalangala	Kibale	Mabira	Malabigambo
Itwara	0.000					
Kalangala	0.000	0.000				
Kibale	0.000	0.000	0.000			
Mabira	0.000	0.000	0.000	0.002		
Malabigambo	0.000	***0.858***	0.000	0.000	0.000	
Zoka	0.000	0.000	0.000	0.000	0.000	0.000

Aridity index (AI)

	Budongo	Itwara	Kalangala	Kibale	Mabira	Malabigambo
Itwara	0.000					
Kalangala	0.000	0.000				
Kibale	***0.213***	0.000	0.000			
Mabira	0.000	0.000	0.000	0.000		
Malabigambo	0.000	***0.690***	0.000	0.000	0.000	
Zoka	0.000	0.000	0.000	0.000	0.000	0.000

Values in the table are *p* values of pairwise comparisons of locations. Significant differences are indicated when $p < 0.05$ and non-significant differences are in bold and italics.
