# Supplementary material for: Genetic diversity of native and cultivated Ugandan Robusta coffee (Coffea canephora Pierre ex A. Froehner): Climate influences, breeding potential and diversity conservation
Source: PLoS One. 2021 Feb 8;16(2):e0245965. doi: 10.1371/journal.pone.0245965 (PMC7870046; doi:10.1371/journal.pone.0245965)

Supplementary Figure S6. Neighbour-joining dendrogram of Ugandan wild *C. canephora* material from the southercenter (SC) population (Mabira, Kalangala and Malabigambo forests) together with cultivated material maintained in Kituza and Kawanda collections (in red) and ferals (in light blue). Individuals representative of other genetic groups (E, R) from the whole species diversity are also presented for reference. Some samples from SC-pop and from the Kituza and Kawanda collections are intermixed with material from genetic group E or R, suggesting that some cultivated material in Uganda was sourced from the Democratic Republic of the Congo (DRC). Each Ugandan accession is labeled and detailed in Supplementary Table S1.

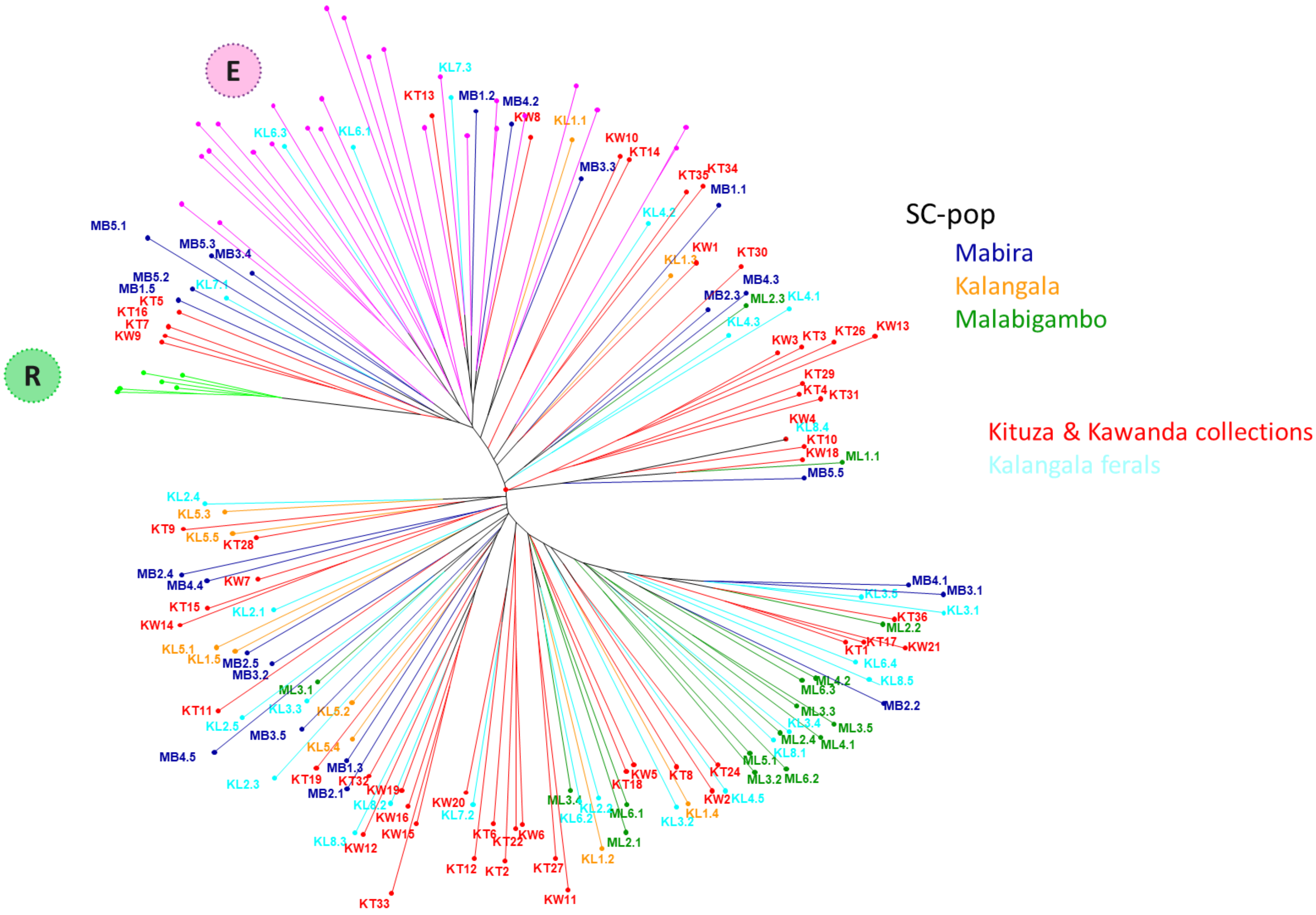

Supplement: S6 Fig — (PDF) [file pone.0245965.s006.pdf]
